# RNA Interference as a Prospective Tool for the Control of Human Viral Infections

**DOI:** 10.3389/fmicb.2018.02151

**Published:** 2018-09-11

**Authors:** Alesia Levanova, Minna M. Poranen

**Affiliations:** Molecular and Integrative Biosciences Research Programme, Faculty of Biological and Environmental Sciences, University of Helsinki, Helsinki, Finland

**Keywords:** RNA interference, antiviral siRNA, antiviral shRNA, siRNA production, siRNA delivery

## Abstract

RNA interference (RNAi), which is mediated by small interfering RNAs (siRNAs) derived from viral genome or its replicative intermediates, is a natural antiviral defense in plants, fungi, and invertebrates. Whether RNAi naturally protects humans from viral invasion is still a matter of debate. Nevertheless, exogenous siRNAs are able to halt viral infection in mammals. The current review critically evaluates the production of antiviral siRNAs, delivery techniques to the infection sites, as well as provides an overview of antiviral siRNAs in clinical trials.

## RNA interference as antiviral defense

The term RNA interference (RNAi) is used to describe gene silencing at the mRNA level guided by small complementary non-coding RNA species. There are several classes of RNAi mediators, one of which, namely small interfering RNAs (siRNAs), facilitates antiviral immunity in plants, fungi, and invertebrates (Ding et al., 2004). The source of siRNAs during infection is viral double-stranded RNA (dsRNA), which is cleaved by cytoplasmic RNAse III family enzyme Dicer into 19–27 base pair (bp) long molecules with a perfectly complementary middle region and 2-nt overhangs on both 3′ ends. These siRNAs are incorporated into a multiprotein RNA-induced silencing complex (RISC). Following the strand separation, the antisense strand guides the RISC to recognize and cut target RNA transcripts (Fire et al., 1998; Elbashir et al., 2001; Macrae et al., 2006).

Whether RNAi is a functional antiviral pathway in mammals is still contentious (tenOever, 2017), since production of siRNA molecules from long dsRNAs cannot be explicitly demonstrated in mammalian cells due to the fact that dsRNA longer than 30 bp triggers activation of interferon (IFN) response (Minks et al., 1979; Elbashir et al., 2001) which shuts down the natural RNAi (Seo et al., 2013). However, mammalian cells do possess all the components of evolutionary conserved RNAi machinery (Shabalina and Koonin, 2008) that can be harnessed to inhibit the expression of cognate mRNA by exogenous siRNA molecules (Elbashir et al., 2001). The antiviral potential of siRNAs was first demonstrated against respiratory syncytial virus (RSV; Bitko and Barik, 2001) and thereafter numerous studies describing antiviral activity of siRNAs against viruses with DNA and RNA genomes *in vitro* and *in vivo* have been published (Gitlin et al., 2002; Jacque et al., 2002; Ge et al., 2003; Kapadia et al., 2003; Randall et al., 2003; Morrissey et al., 2005; Kumar et al., 2008; Geisbert et al., 2010; Paavilainen et al., 2017; Villegas et al., 2018). RNAi-based drugs appear to be a viable option to treat severe viral infections, against which effective vaccines or specific cure is not yet available, such as Ebola virus or emerging viruses. Furthermore, siRNAs are likely to become a valuable alternative to treat debilitating chronic infections caused by human immunodeficiency virus (HIV) and hepatitis B virus (HBV). Current care for chronic hepatitis B infection is a combination of nucleos(t)ide analogs and interferon (Su and Liu, 2017), while combination antiretroviral therapy, which targets viral enzymes as well as cellular entry receptors is used to treat HIV infection (Cihlar and Fordyce, 2016). However, these treatments are of limited effectiveness, toxic, impose the risk of developing drug resistance, and life-long since they only suppress the virus and do not lead to eradication of infection. Conversely RNAi-based drugs have a potential to achieve a functional cure and discontinue antiviral therapy.

## Production of RNA molecules for RNAi

### Selection of target sequences for RNAi

The first step in production of antiviral siRNAs is *in silico* selection of highly conservative sequences in the targeted virus genome in order to achieve strong antiviral activity and avoid off-target effects. Specificity filters are used to exclude sequences with close similarity to mRNAs of human and model animal transcriptomes. Additionally, a number of sequence- and structure-based algorithms are applied to select the functionally most potent siRNA sequences (Reynolds et al., 2004; Tafer, 2014). However, this step is not needed when pools of siRNAs covering large regions of conserved sequences are used (see Enzymatic generation of siRNAs).

### Production of siRNAs

Three approaches have been developed to generate antiviral siRNAs: (1) chemical synthesis; (2) enzymatic production; and (3) *in vivo* expression from siRNA expression cassette or vector.

#### Chemical synthesis of RNA molecules

Molecules of ssRNA are produced by automated solid-phase synthesis employing 2′-hydroxyl protecting groups that provide ribonucleoside phosphoramitides (Beaucage, 2008). Following the synthesis step, cognate ssRNAs are hybridized to form RNA duplexes. The accuracy of chemical RNA synthesis is often compromised, resulting in products of varying length and sequence. Therefore, each ssRNA must be tested by matrix-assisted laser desorption-ionization mass spectrometry, and produced siRNAs are analyzed by non-denaturing gel or capillary electrophoresis to confirm proper annealing of the strands^1^. Albeit the cost of synthesis per nucleotide has decreased dramatically over the past few years, chemical synthesis is still far from an economically sound approach. However, if the cost is not a limiting factor, this method is so far indispensable for applications that require large amounts of ultrapure siRNA molecules with defined sequence (e.g., clinical trials).

#### Enzymatic generation of siRNAs

**Figure 1:**
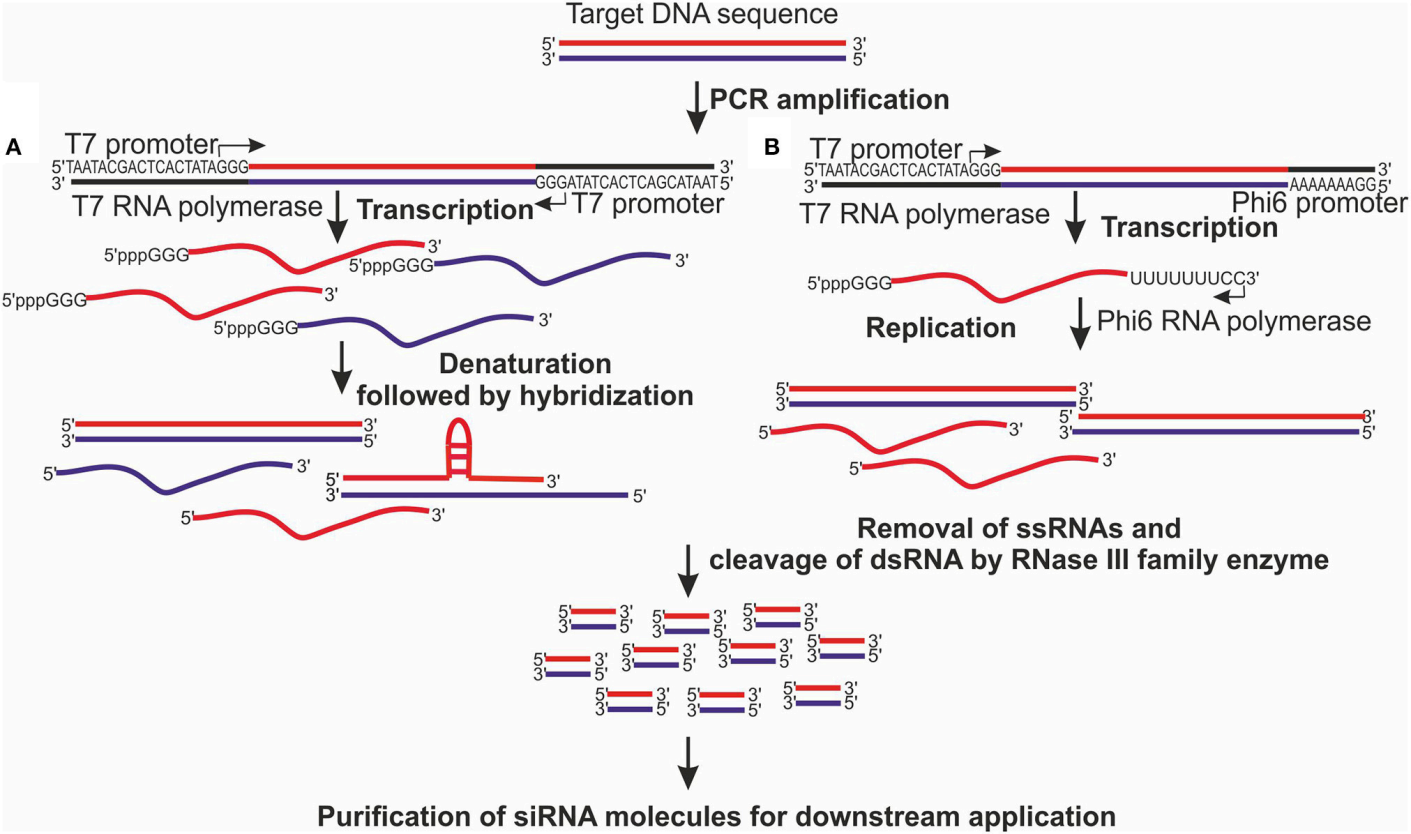
Enzymatic production of siRNA pools. **(A)** Target-specific DNA template is generated by PCR with forward and reverse primers containing at their 5′ ends promoter sequence for T7 DNA-dependent RNA polymerase. T7 polymerase transcribes purified DNA into single-stranded RNA (ssRNA) molecules, which are subsequently heated at 75°C for 5 min to denature secondary and tertiary ssRNA structures and to allow proper strand annealing. Nevertheless, long ssRNA molecules are prone to formation of branched structures and hairpins, which are difficult to denature and which prevent proper hybridization, and decrease the amount of full-length double-stranded RNAs (dsRNAs). **(B)** DNA template is generated by PCR with forward primer containing a promoter sequence for T7 polymerase and reverse primer comprising a promoter sequence for Phi6 RNA-dependent RNA polymerase. The resulting product is purified and a single-tube reaction is set up, where T7 polymerase transcribes DNA into ssRNA and Phi6 polymerase replicates the second strand of the RNA molecule. This approach results in a higher yield of full-length, biologically active dsRNA molecules compared to that where annealing step is used to generate dsRNA. **(A,B)** The generated dsRNAs are purified and cleaved by RNase III family enzyme into siRNA pool, which is processed according to the requirement of the downstream application.

The main advantages of enzymatic production of siRNAs are low-cost and short preparation time. Therefore, this method can be applied for screening of the most potent siRNA sequences. Traditionally enzymatic production of siRNAs and longer dsRNAs has been based on *in vitro* transcription of DNA templates containing T7 polymerase promoter (Figure 1). T7 DNA-dependent RNA polymerase (DdRp) transcribes target DNA molecules in their sense and antisense orientation followed by the annealing step (Donzé and Picard, 2002; Sohail et al., 2003). The leader sequence added by T7 DdRp to the 5′ end of the synthesized ssRNA can be cleaved off by deoxyribozyme, while random nucleotides, that T7 DdRp might add to the 3′ end of ssRNA, do not have any considerable negative effect on siRNAs efficacy and safety (Sohail et al., 2003). The bacteriophage polymerases also incorporate 5′ triphosphates to their transcripts, which can induce a significant IFN response (Kim et al., 2004). However, treatment of synthesized ssRNA with alkaline phosphatase abrogates the IFN induction. The main disadvantage of T7 DdRp-based dsRNA production is a rather limited dsRNA yield because during hybridization step a significant amount of biologically inactive dsRNA is generated, especially in the case of long ssRNAs, due to formation of tertiary structures that prevent annealing of complementary sequences (Figure 1).

To avoid the drawbacks of inefficient annealing step, our laboratory has developed a single-tube dsRNA synthesis platform, where ssRNA generated by T7 DdRp is immediately used by bacteriophage Phi6 RNA-dependent RNA polymerase (RdRp) to synthesize a complementary RNA strand starting from the very 3′ end (Aalto et al., 2007; Figure 1). Phi6 RdRp is a highly processive enzyme lacking template specificity and providing an opportunity to produce dsRNA molecule of virtually any length from any heterologous template (Makeyev and Bamford, 2000). Besides T7 DdRp and Phi6 RdRp, other viral polymerases such as DdRp from bacteriophages T3, SP6, cyanophage Syn5 (Zhu et al., 2015), and noroviral RdRp (Rohayem et al., 2006) can be used for RNA synthesis *in vitro*. The generated dsRNA molecules can be subsequently digested with either bacterial RNase III (Yang et al., 2002), recombinant eukaryotic Dicer (Gimenez-Barcons et al., 2007; Romanovskaya et al., 2012), or RNase T1 (Hannus et al., 2014) to get a pool of target-specific siRNA molecules.

Enzymatic approach for siRNAs production allows to generate siRNA pool against any virus in a relatively short time, which is essential in case of a sudden virus outbreak. A robust and fairly fast protocol for the purification of enzymatically-produced siRNAs has been developed in our laboratory, where siRNAs obtained by *Giardia* Dicer digestion are purified by anion-exchange chromatography on monolithic QA column followed by desalting on Sephadex G25 column (Romanovskaya et al., 2013). The siRNAs obtained are of high purity and safe for animals (Paavilainen et al., 2017).

A diverse siRNA pool derived from a long fragment of viral genome, which mimics the natural RNAi-based antiviral defense, is more protective than a single-site siRNA. Even RNA viruses with high mutation potential can be effectively inhibited with a mixture of siRNAs and escape mutants are not generated that easily compared to a single siRNA (Gitlin et al., 2005). Importantly, each siRNA species in a pool is present at very low concentration diluting off-target effects below detection limit. A number of studies demonstrated that siRNA pools generated enzymatically are highly effective in silencing of the target genes without causing obvious off-target effects (Aalto et al., 2007; Nygårdas et al., 2009; Romanovskaya et al., 2012; Hannus et al., 2014; Paavilainen et al., 2016, 2017). Nevertheless, despite numerous benefits of using siRNA pools, there are no enzymatically-produced siRNA mixtures in clinical trials.

A possibility to generate dsRNA in mammalian or bacterial cells, using viral RNA polymerases, has been demonstrated, which potentially allows to scale-up the production at low cost (Aalto et al., 2007; Heninger and Buchholz, 2007; Huang and Lieberman, 2013; Enayati et al., 2016). However, these systems still need further elaboration and most probably will not have clinical applications but, for instance, can be used in agriculture (Tenllado et al., 2003; Niehl et al., 2018).

#### In vivo expression from siRNA expression vector

Molecules of siRNAs can also be produced by Dicer cleavage of small hairpin RNA (shRNA) transcribed in a cell from an expression cassette containing a polymerase III promoter (U6 or H1), a DNA template of desired shRNA sequence, and transcription stop signal (Brummelkamp et al., 2002). The expression cassette can be integrated into a plasmid or viral vector and thus delivered into cells. The main advantage of siRNA expression vectors is that they are suitable for long-term applications.

## Delivery of siRNAs for clinical applications

Depending on the target tissue, siRNA therapeutics can be administered either locally or systemically via intravenous injection. However, unprotected siRNAs are prone to rapid degradation by ubiquitous endo- and exonucleases and they are undetectable in the blood already 10 min after administration (DeVincenzo et al., 2008). Due to a strong anionic charge of the phosphate backbone, siRNAs cannot passively diffuse through negatively charged cellular membranes. Moreover, siRNA molecules can be sensed by the cellular receptors and induce IFN response or other off-target effects (Anderson et al., 2008; Sioud, 2009; Olejniczak et al., 2011). Several approaches have been developed to enhance siRNA stability and promote its cellular uptake. The most widely used approach involves introduction of chemical modifications to ribose sugar, phosphate linkage, or base of nucleotide (Kaczmarek et al., 2017). RNA can be easily modified during chemical synthesis. Furthermore, bacteriophage polymerases (e.g., DdRps from T7 and Syn5) are also able to utilize modified NTPs for RNA synthesis (Zhu et al., 2015; Sun and Riggs, 2017). Modifications of the sugar backbone at the 2′-position of the ribose ring [2′-O-methyl (2′-OMe), 2′-deoxy, and 2′-fluor] not only have no impact on gene silencing indicating that 2′-OH group is dispensable for functional RNAi pathway, but demonstrate increased siRNA plasma stability and consequently enhanced *in vivo* efficacy with reduced off-target effects (Chiu and Rana, 2003). Moreover, these modification render siRNAs unrecognizable for immune system (Morrissey et al., 2005).

RNA delivery to the target cells can be achieved by viral and non-viral vectors. For the delivery to animals and humans, adeno-associated virus (AAV) vector is the first choice since it has been proved to be non-toxic, non-pathogenic, easy to produce, and it does not integrate into human genome (Naso et al., 2017). Of the non-viral vectors, nanoparticles comprised of cationic polymers (poly-L-lysine, polyamidoamine, polyethyleneimine, chitosan) or lipids are the best studied delivery vehicles (Kaczmarek et al., 2017). In addition to nanoparticles, direct conjugates of bioactive ligands to siRNA molecules can facilitate their entry to the cell. Once siRNA is complexed with positively charged polymer or lipid molecules, it can approach cell membrane close enough to be internalized via micropinocytosis or clathrin-dependent pathway (Pozzi et al., 2014). If siRNA is conjugated with specific ligands (e.g., antibodies) that recognize receptor molecules on a particular cell type, it can be taken up by receptor-mediated endocytosis. In this case endosome escape agents must be applied to facilitate siRNA transport to cytosol, where it can be used by RNAi machinery (Dominska and Dykxhoorn, 2010).

Topical delivery of antiviral siRNA seems to be a viable approach for mucosal surfaces and has been used to inhibit herpes simplex virus (HSV) and RSV in animal models (Bitko et al., 2005; Palliser et al., 2006; Alvarez et al., 2009; Paavilainen et al., 2017). In clinical studies, human respiratory tract can be easily achieved by inhalation of an aerosol indicating a plausible administration route for antivirals against respiratory viruses. For a detailed review on the approaches to siRNA delivery please refer to (Musacchio and Torchilin, 2013; Kaczmarek et al., 2017).

## Antiviral RNAi-based therapeutics

The first siRNA with documented effect in humans was ALN-RSV01, a 19 bp RNA duplex with two (2'-deoxy) thymidine overhangs on both 3′ ends to prevent its nuclease degradation (Elbashir et al., 2001; Alvarez et al., 2009). ALN-RSV01 targets a highly conserved region in the mRNA of RSV nucleocapsid protein. The activity of naked ALN-RSV01 siRNAs has been tested in adults experimentally infected with wild-type RSV. The siRNA was applied daily in the form of nasal spray, 2 days before and 3 days after RSV infection. Intranasal ALN-RSV01 administration was safe and well tolerated and resulted in a 38% decrease in the number of infected people (DeVincenzo et al., 2010). Furthermore, ALN-RSV01 has been shown to reduce the risk of bronchiolitis obliterans syndrome in RSV-infected lung transplant patients in Phase 2 randomized, double-blind, placebo-controlled trials (Zamora et al., 2011; Gottlieb et al., 2016). Alnylam Pharmaceuticals had been developing a nasally administered formulation of asvasiran sodium (ALN-RSV01). However, in 2014 clinical trials were suspended for undisclosed reason. In our opinion, this could be related to the emergence of drug resistant viruses, which are easily generated if only a single-site siRNA molecule is used.

At least eight anti-HBV siRNA formulations have been in clinical trials (Table 1; Flisiak et al., 2018). Nevertheless the results from these trials are mostly reported at different scientific meetings instead of publications in peer-reviewed journals and, therefore, the detailed information is scarce. In this paragraph we will focus on siRNAs developed by Arrowhead Pharmaceuticals due to the abundance of the published data available for analysis. The first generation anti-HBV siRNA pool, ARC-520, was comprised of two synthetic siRNAs targeting the common region at the 3′ end of all HBV transcripts from episomal HBV DNA. The siRNAs were conjugated to cholesterol, which facilitates the cellular uptake and protects from degradation by serum RNAses (Schroeder et al., 2010). These conjugates were intravenously co-injected with polymer-based system (Rozema et al., 2007), which was composed of amphipathic membrane active peptide, required for endosome escape, and N-acetylgalactoseamine, responsible for hepatocyte-specific delivery via asialoglycoprotein receptor that is highly expressed on the surface of hepatocytes (Wooddell et al., 2013; Nair et al., 2014). ARC-520 tolerability and pharmacokinetics has been studied in healthy volunteers with no indicated adverse effects (Schluep et al., 2017). However, the data from a phase II clinical trials (Wooddell et al., 2017) have shown that a number of patients had only minimal response to ARC-520 treatment due to the integration of the HBV genome into the host DNA, which led to the loss of the target sites for ARC-520 (Wooddell et al., 2017). Therefore, the next formulation, ARC-521, in addition to already validated siRNA sequences included siRNA targeting viral mRNA expressed from the integrated HBV genome. Although siRNAs themselves were well tolerated in humans, endosome escape agent caused some toxicity in experimental animals. Therefore, Arrowhead Pharmaceuticals has switched to the development of the second generation siRNA-based drug ARO-HBV, in which the formulation of excipient has been changed. The preclinical studies have shown that APO-HBV has significant antiviral activity, and its doses up to 300 mg/kg given weekly are well tolerated in laboratory animals (Wooddell et al., 2018)^2^.

**Table 1:** Antiviral siRNAs in clinical trials.

**Target virus**	**Drug details**	**Delivery system**	**Administ-ration**	**Clinical Trials Identifier^a^**	**Current status**	**Sponsor**	**Reference**
**Respiratory syncytial virus (RSV)**	**ALN-RSV01**, a single site siRNA targeted to nucleocapsid gene	Naked siRNA	Intranasal	NCT00496821 NCT00658086 NCT01065935	Phase 2, 2b, completed	Alnylam Pharmaceuticals	Zamora et al., 2011
**Hepatitis B virus (HBV)**	**NUC B1000**, four shRNAs, of which one is targeted to polymerase gene and three are complementary to the gene encoding hepatitis B surface antigen (HBsAg)	Plasmid DNA in cationic lipids	Intravenous	NA	Phase 1	Nucleonics	Gish et al., 2011
	**ARC-520**, 2 synthetic siRNAs mapping to the common region at the 3' end of all HBV transcripts from episomal DNA	Polymer-based system (Dynamic PolyConjugate, DPC)	Intravenous	NCT02738008 NCT02349126 NCT02604199 NCT02604212 NCT02577029 NCT02065336 NCT02452528	Phase 2, terminated	Arrowhead Pharmaceuticals	Wooddell et al., 2017
	**ARC-521**, two siRNA as in ARC-520 and one siRNA targeted to mRNA transcripts from integrated viral genome	DPC	Intravenous	NCT02797522	Phase 1, terminated	Arrowhead Pharmaceuticals	NA
	**ARO-HBV**, siRNA as in ARC-521	Modified delivery system (TRIM)	Subcutaneous	NCT03365947	Phase 1/2a, recruiting	Arrowhead Pharmaceuticals	NA
	**ARB-1467** (TKM-HBV), three siRNAs that target all four HBV transcripts, in combination with nucleos(t)ide analog	Lipid nanoparticles (LNP)	Intravenous injection alongside steroids	NCT02631096	Phase 2, completed	Arbutus Biopharma	Streinu-Cercel et al., 2017
	**ARB-1740**, a different set of three siRNAs that target all four HBV transcripts in combination with nucleos(t)ide analog	The same LNPs as for ARB-1467	Intravenous	ACTRN12617000557336^b^	Phase 1	Arbutus Biopharma	NA
	**AB-729**, more detailed information is NA	N-acetylgalactoseamine (GalNac)-siRNA conjugates	Subcutaneous	NA	Phase 1 is starting in 2019	Arbutus Biopharma	NA
	**ALN-HBV**, a single-site chemically modified siRNA, that targets a conserved region present in all viral transcripts	GalNac-siRNA conjugates	Subcutaneous	NCT02826018	Phase 1/2, terminated	Alnylam Pharmaceuticals	NA
**Human immuno-deficiency virus (HIV-1)**	**LVsh5/C46** (Cal-1), a combination of the shRNA for downregulation of CCR5 and the HIV-1 fusion inhibitor, C46	Self-inactivating lentiviral vector based on HIV-1 backbone	Infusion transduced autologous hematopoietic stem cells or CD4+ T cells	NCT01734850 NCT02390297 NCT02378922	Phase1/2, completed long term follow-up Phase 1, suspended	Calimmune, Inc Fred Hutchinson Cancer Research Center	Symonds et al., 2016
	**rHIV7-shI-TAR-CCR5RZ**, a combination of shRNAs targeted to tat/rev common exon, trans-activation response RNA (TAR) decoy, and anti-CCR5 ribozyme	Recombinant lentivirus vector HIV7	Infusion of transduced autologous hematopoietic stem cells (CD34+)	NCT01961063 NCT00569985 NCT02337985	Phase 1, active	City of Hope Medical Center and National Cancer Institute (NCI)	DiGiusto et al., 2010
	a cocktail of shRNAs targeting CCR5 and HIV genome	Lentivirus vector	Infusion of transduced autologous hematopoietic stem cells (CD34+)	NCT03517631	Phase 1, recruiting	Shanghai Public Health Clinical Center and Kanglin Biotech	NA
	a shRNA targeted to CCR5, TRIM5alpha, and TAR decoy	Lentivirus vector	The same as above	NCT02797470	Phase 1/2, recruiting	AIDS Malignancy Consortium	NA
**Hepatitis C virus (HCV)**	**TT-034**, three shRNAs targeted to HCV genome	Adeno-associated viral vector 8	Intravenous	NCT01899092 NCT02315638	Phase1/2	Benitec Biopharma	NA
**Zaire ebolavirus (ZEBOV)**	**TKM-100201** (TKM-EBOV-001), three chemically modified siRNAs to target regions of the ZEBOV mRNAs transcribed from polymerase L, membrane-associated viral protein 24 (VP24), and polymerase cofactor VP35 genes	LNPs	Intravenous	NCT01518881	Phase 1, terminated	Arbutus Biopharma (former Tekmira Pharmaceutical)	NA
	**TKM-100802** (TKM-EBOV-002), two siRNAs to target regions of the viral polymerase L and VP35	LNPs	Intravenous	NCT02041715	Phase 1, terminated	Arbutus Biopharma	Kraft et al., 2015
	**TKM-130803**, comprised of the same siRNA as TKM-100802 but anti-L siRNA contains one nucleotide substitution and anti-VP35 siRNA two nucleotide substitutions to ensure specificity to the West African Makona strain of EBOV	New formulation of LNPs	Intravenous	NA	Single-arm phase 2	Arbutus Biopharma	Dunning et al., 2016

*NA – information is not available*.

a *The information is obtained from the web page https://clinicaltrials.gov/, if not indicated otherwise*.

b *The information is obtained from the web page http://www.anzctr.org.au/*.

In spite of tremendous efforts put into the development of treatment of infection with HIV, currently only one patient has attained functional recovery, and no HIV can be detected in his blood or other tissues tested (Hütter et al., 2009). This HIV-infected person developed an acute myeloid leukemia and was subjected to hematopoietic stem cell transplantation in 2007. Notably, a donor had a 32 bp deletion in both alleles coding for chemokine receptor CCR5 (Hütter et al., 2009), and the majority of HIV-1 viruses utilize CCR5 as a co-receptor to enter CD4+ cells (Alkhatib, 2009). A number of phase I/II clinical studies (Table 1) try to mimic the conditions under which the functional recovery was achieved and transduce autologous hematopoietic CD34+ stem cells with lentiviral vectors carrying multiplexed shRNA which target not only conserved regions in HIV genome, but also the host CCR5 gene. For a detailed review of RNAi-based therapies against HIV refer to (Bobbin et al., 2015; Scarborough and Gatignol, 2017).

A pool of three chemically modified siRNAs preventing synthesis of Zaire ebolavirus (ZEBOV) polymerase, viral proteins 24, and 35 completely protected rhesus macaques from lethal infection (Geisbert et al., 2010). This siRNA formulation with some modifications (Table 1), lately referred to as TKM-Ebola, was subsequently applied to treat humans during the ZEBOV outbreak in West Africa in 2013–2016. However, TKM-Ebola did not improve survival, which might be connected to a poor design of the clinical trials and inclusion of only terminally sick patients with high viral loads (Cross et al., 2018).

Two clinical trials have been initiated to assess the efficacy and safety of shRNA-based TT-034 therapeutics, which was created for chronic hepatitis C treatment and delivered to hepatocytes by AAV vector. TT-034 was shown to be safe and well-tolerated. However, in February 2016 Benitec Biopharma decided to discontinue hepatitis C program due to low commercial opportunities^3^.

In conclusion, the development of RNAi-based therapeutics is still in its early stage and has experienced numerous pitfalls. Nonetheless, it has already been demonstrated that siRNAs can effectively inhibit the replication of various viruses despite different mechanisms they evolved to resist the pressure imposed by immune system and antiviral drugs. However, the possibility of generation of escape mutants, recently discovered inhibitors of RNAi in human viruses (Fabozzi et al., 2011; Qiu et al., 2017), and complex interactions between RNAi and interferon pathways (Kok et al., 2011; Seo et al., 2013; Maillard et al., 2016) must be taken into consideration when designing new antiviral siRNA molecules. Furthermore, there are still unresolved issues with safe and efficient delivery of siRNAs to the target tissues and cells, which can be unraveled by fundamental research in the area.

## Author contributions

AL and MMP conceptualization, AL manuscript draft, AL and MMP manuscript revision.

### Conflict of interest statement

The authors declare that the research was conducted in the absence of any commercial or financial relationships that could be construed as a potential conflict of interest.

## Footnotes

^1^https://www.thermofisher.com/us/en/home/references/ambion-tech-support/rnai-sirna/tech-notes/five-ways-to-produce-sirnas.html.

^2^http://ir.arrowheadpharma.com/static-files/9de6c80f-459a-419a-87a0-f82125d0196b.

^3^https://www.prnewswire.com/news-releases/update-on-tt-034-hepatitis-c-clinical-trial-300226692.html.
